# Antibacterial Activities and Antibacterial Mechanism of *Polygonum cuspidatum* Extracts against Nosocomial Drug-Resistant Pathogens

**DOI:** 10.3390/molecules200611119

**Published:** 2015-06-16

**Authors:** Pai-Wei Su, Cheng-Hong Yang, Jyh-Ferng Yang, Pei-Yu Su, Li-Yeh Chuang

**Affiliations:** 1 Institute of Biotechnology and Chemical Engineering, I-Shou University, Kaohsiung 840, Taiwan; E-Mails: czscientist@gmail.com (P.-W.S.); jfyang@isu.edu.tw (J.-F.Y.); evangelion_0723@yahoo.com.tw (P.-Y.S.); 2 Department of Electronic Engineering, National Kaohsiung University of Applied Sciences, Kaohsiung 807, Taiwan; E-Mail: chyang@cc.kuas.edu.tw

**Keywords:** *Polygonum cuspidatum*, antibacterial activity, clinical drug-resistant bacteria

## Abstract

Recently, drug resistance due to the extensive abuse and over-use of antibiotics has become an increasingly serious problem, making the development of alternative antibiotics a very urgent issue. In this study, the Chinese herbal medicine, *Polygonum cuspidatum*, was extracted with 95% ethanol and the crude extracts were further purified by partition based on solvent polarity. The antimicrobial activities of the extracts and fractions were determined by the disk diffusion and minimum inhibitory concentration (MIC) methods. The results showed that the ethyl ether fraction (EE) of the ethanol extracts possesses a broader antimicrobial spectrum and greater antimicrobial activity against all of the tested clinical drug-resistant isolates, with a range of MIC values between 0.1–3.5 mg/mL. The active extract showed complete inhibition of pathogen growth and did not induce resistance to the active components. In addition, according to scanning electron microscope observations, EE resulted in greater cell morphological changes by degrading and disrupting the cell wall and cytoplasmic membrane, whereby ultimately this cell membrane integrity damage led to cell death. In conclusion, the EE extracts from *Polygonum cuspidatum* may provide a promising antimicrobial agent for therapeutic applications against nosocomial drug-resistant bacteria.

## 1. Introduction

In recent years, with increasing technology and medical knowledge, people have become more aware of the quality of medical care. In Taiwan, medical treatment lacks a better control on long-term antibiotics usage to treat various diseases. The livestock and aquaculture industry have used antibiotics in animal feed excessively to prevent infectious diseases, leading bacteria to produce drug resistance. Drug resistant bacteria spread among different hosts, which in turn leads to drug resistance gene transfer within different drug-resistant bacteria, resulting in the emergence of multiple drug-resistant bacteria [1,2,3]. According to the clinical epidemiology analysis reports, *Staphylococcus aureus*, *Pseudomonas aeruginosa* and *Acinetobacter baumannii* has become the most common nosocomial infection and drug resistant strains, with infection rates as high as 50% [4]. These clinical drug-resistant strains have increased the treatment difficulty or even led to the outbreak of severe nosocomial infections, and this lack of an effective therapy has led to an urgent search for novel antibacterial substances.

Chinese herbal medicines have a long history of important roles in the treatment of many diseases. However, most herbal medicines have not been developed as commercial drugs due to fact their dosages and mode of action have not been clarified, despite the fact that compared with most of the currently used commercial antibiotics, natural antibacterial ingredients would appear to have the benefits of lesser side effects and toxicity as well as a higher stability [5,6,7].

*Polygonum cuspidatum* (called Huzhang), belongs to the Polygonaceae family and is wildly distributed in Asia and North America. The roots of *Polygonum cuspidatum* are officially listed in the Chinese Pharmacopoeia used as a traditional Chinese medicine for the treatment of various inflammatory diseases, hepatitis, tumors, and diarrhea [8]. It has gained much notoriety in Europe and North America (where it is known as Mexican bamboo, Japanese bamboo, or Japanese knotweed) as an invasive neophyte (introduced after 1500), due to its virtually indestructible growth as a fast-growing, robust perennial herb that emerges early in the spring and forms dense thickets of up to 9 feet in height [9]. According to the literature reports, many phenolic compounds, included resveratrol, piceid and emodin, have been verified to possess various biological activities, among which, resveratrol and piceid have revealed antioxidant, anti-iflammatory, anti-cancer, anti-aging and cardioprotective properties [10]. Emodin showed anti-inflammatory, antibacterial and antineoplastic activity [11]. 

This study is targeted at the common drug-resistant strains of nosocomial infections, *Staphylococcus aureus*, *Acinetobacter baumannii* and *Pseudomonas aeruginosa*, to explore the antimicrobial activity and antibacterial mechanism of the extracts of *Polygonum cuspidatum*, hoping to discover a new generation of antibiotic substitutes against the clinical drug resistant pathogens.

## 2. Results

### 2.1. Antimicrobial Activity of the Polygonum cuspidatum Extracts by the Disc Diffusion Method

Six clinical antibiotic-resistant isolates, including two of *Staphylococcus aureus* (Sa225, Sa2805), *Pseudomonas aeruginosa* (Pa4016, Pa1347) and *Acinetobacter baumannii* (Ab2260, Ab3394), as well as four standard strains, including *S. aureus* ATCC6538P, *P. aeruginosa* ATCC29260, *P. aeruginosa* ATCC27853, and *A. baumannii* ATCC19606 , were selected for the antibacterial analysis. As shown in Table 1, the disc diffusion analysis results (30 μL/disc; 0.1 g/mL dissolved in DMSO) revealed that the ethanol crude extracts of *Polygonum cuspidatum* showed antibacterial activity against the test strains of *S. aureus*, *A. baumannii* and *P. aeruginosa* with average inhibition zones of 21.00 mm, 15.50 mm and 11.50 mm, respectively. The crude extract showed more significant antibacterial activity against the strains of *S. aureus* compared with the other test strains. Among all of the partitioned fractions, the ethyl ether (EE) and ethyl acetate (EA) fractions showed a higher antimicrobial activity against all of the test strains. The EE extract revealed the most significant antibacterial activity and broad antimicrobial spectrum against the strains of *S. aureus*, *A. baumannii* and *P. aeruginosa*, with average inhibition zones of 26.00 mm, 20.33 mm and 17.00 mm, respectively. The EA extract presented a slightly lower antimicrobial activity than the EE extract. The negative control, DMSO, did not show any inhibition zone against all the test strains. The positive control, tetracycline, showed an average inhibition zone of 29.67 mm, 15.00 mm and 15.00 mm against the strains of *S. aureus*, *A. baumannii* and *P. aeruginosa*, respectively.

**Table 1:** Antimicrobial activity of the *Polygonum cuspidatum* extracts against the test microorganisms.

Test Strains *	Disc Inhibition Zone (DIZ: mm)					
	Crude Extract	*n*-Hexane	Chloroform	Ethyl Ether	Ethyl Acetate	Aqueous
Sa6538p	20.00	17.00	18.00	24.00	23.00	14.00
Sa335	22.00	20.00	20.00	28.00	27.00	18.00
Sa2803	21.00	20.00	20.00	26.00	26.00	12.00
Ab19606	16.00	9.00	NA	20.00	12.00	NA
Ab2260	NA	9.00	9.00	18.00	11.00	9.00
Ab3394	15.00	9.00	9.00	23.00	16.00	NA
Pa29260	NA	NA	NA	13.00	10.00	NA
Pa4016	NA	9.00	9.00	22.00	15.00	NA
Pa1347	11.00	9.00	9.00	20.00	14.00	NA
Pa27853	12.00	NA	NA	13.00	10.00	NA

* Sa: *Staphylococcus aureus*; Ab: *Acinetobacter baumannii*; Pa: *Pseudomonas aeruginosa*; NA: No Activity.

### 2.2. Minimum Inhibitory Concentration (MIC)

As the disc diffusion analysis results showed that the EE and EA fractions (30 μL/disc; 0.1 g/mL dissolved in DMSO) revealed a significant antimicrobial activity against the test pathogens, the crude extract and both partitioned fractions with high activity were thus selected for the determination of minimum inhibitory concentration (MIC). As summarized in Table 2, the MIC of the EE fraction indicated a highest antimicrobial activity against all of the pathogens in the average range of 0.2–1.63 mg/mL. The Gram positive strain (*S. aureus*) with MIC range of 0.2–1.00 mg/mL was more susceptible to the test extracts than the Gram negative strains (*A. baumannii* and *P. aeruginosa*) with MIC ranges of 0.75–19.50 mg/mL. Comparing the antimicrobial activities of EE fraction and crude extracts, the EE fraction displayed about 3–10 fold higher antimicrobial activity than the crude extracts. Regarding the antimicrobial spectrum, most of the test strains, included standard strains and clinical drug-resistant isolates, showed similar susceptibility to the extracts/fractions, except the *P. aeruginosa* strains; both clinical isolates of *P. aeruginosa* (Pa4016 and Pa1347) showed more susceptible to the extracts than the two standard strains of *P. aeruginosa* (Pa29260 and Pa27853).

**Table 2:** The Minimum Inhibitory Concentration (MIC) of the *Polygonum cuspidatum* extracts against the test microorganisms.

Strains	Minimum Inhibitory Concentration (mg/mL)		
	Ethyl Ether	Ethyl Acetate	Crude Extracts
*Staphylococcus aureus*			
Sa6538p	0.10	1.00	0.38
Sa335	0.25	1.00	0.38
Sa2803	0.25	1.00	0.38
mean ± SD * (*n* = 3)	0.20 ± 0.07	1.00 ± 0.00	0.38 ± 0.00
*Acinetobacter baumannii*			
Ab19606	0.75	2.00	12.00
Ab2260	0.75	3.00	12.00
Ab3394	0.75	3.00	11.00
mean ± SD * (*n* = 3)	0.75 ± 0.00	2.67 ± 0.47	11.67 ± 0.47
*Pseudomonas aeruginosa*			
Pa29260	2.50	2.50	30.00
Pa27853	2.50	3.00	30.00
Pa4016	0.75	2.50	7.00
Pa1347	0.75	3.00	11.00
mean ± SD * (*n* = 4)	1.63 ± 0.88	2.75 ± 0.25	19.50 ± 10.59

Mean ± SD *: mean ± standard deviations for the same species.

### 2.3. Time-Killing Assay

**Figure 1:**
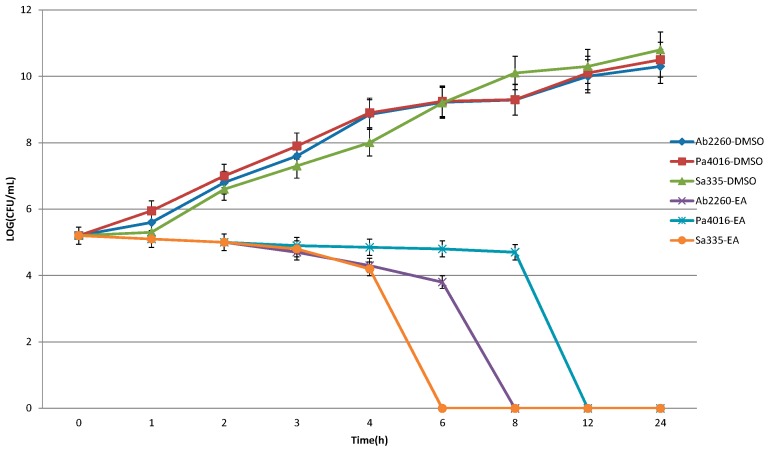
Bactericidal effect of the ethyl acetate fraction of *Polygonum cuspidatum* on the test strains of *S. aureus* 335, *A. baumannii* 2260 and *P. aeruginosa* 4016. A suspension of 3 × 10^5^ CFU/mL of bacterial strains were cultured in the two fold MIC dose of herbal extracts or solvent DMSO. Aliquots were withdrawn, plated on agar, incubated for 16 h and viable colonies were counted.

**Figure 2:**
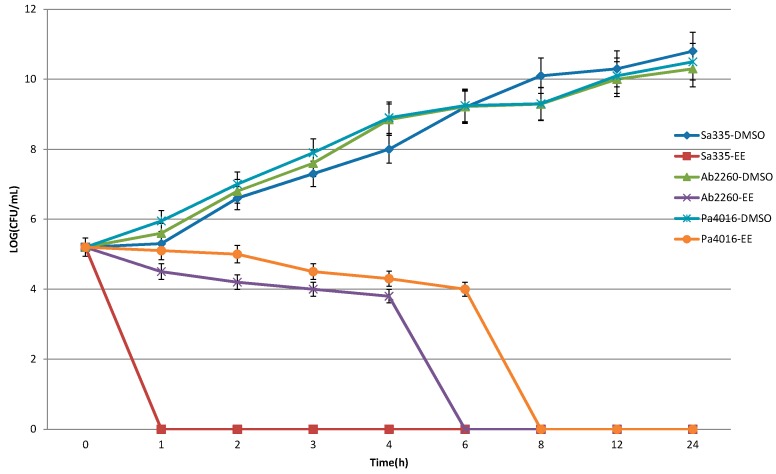
Bactericidal effect of the ethyl ether fraction of *Polygonum cuspidatum* on the test strains of *S. aureus* 335, *A. baumannii* 2260 and *P. aeruginosa* 4016. A suspension of 3 × 10^5^ CFU/mL of antibacterial strains were cultured in the 2-ffold MIC dose of herbal extracts or solvent DMSO. Aliquots (200 μL) were withdrawn, plated on agar, incubated for 16 h and viable colonies were counted.

The bactericidal ability of the partitioned EE and EA fractions with significant antimicrobial activity was determined by time-killing curves. Three clinical isolates from each strain, including *P. aeruginosa* 4016, *S.* *aureus* 335 and *A. baumannii* 2260, were selected for the assay. Figure 1 and Figure 2 show that the time killing curve of negative control, solvent DMSO, revealed that the colonies increased over time, which means DMSO did not possess any bactericidal ability against the test strains. The time killing curve of EA extract revealed a significant bactericidal effect, in which the strains of *P. aeruginosa* 4016, *A. baumannii* 2260 and *S. aureus* 335 were completely killed in 12, 8, and 6 h, respectively. The time killing curve of EE extract showed that the *P. aeruginosa* 4016, *A. baumannii* 2260 and *S. aureus* 335 bacteria counts went from 3 × 10^5^ CFU/mL down to 0 CFU/mL in 8, 6, and 1 h, respectively. All of the sterilization effects of the extracts were maintained for 24 h, thus showing that the *Polygonum cuspidatum* extracts not only possessed antibacterial activities, but also had bactericidal abilities.

### 2.4. Resistance Analysis

The drug induced resistance was determined by subculture of the microorganisms with a sub-MIC concentration of the EE extract for 10 consecutive days. On day 11, the MIC of each subculture was determined. The result showed that the MIC for the test strains, including *P. aeruginosa* 4016, *A. baumannii* 2260 and *S. aureus* 335, were unchanged, demonstrating that the test pathogens would not be expected to induce resistance to the active substances from the EE extract.

### 2.5. Combination Effect of the Active Herbal Fractions with Antibiotics

The synergy effects of the extracts with antibiotics were determined by the disc diffusion method. The strains of *S. aureus* 335, *A. baumannii* 2260 and *P. aeruginosa* 4016 were selected as the test strains; a total of 12 antibiotics, included erytromycin, gentamicin, tetracycline, spectinomycin, piperacillin G, kanamycin, amikacin, clindamycin, ampicillin, cephalosporinin, trimethoprim/sulfamethoxazole, and amoxillin, were used for the synergy effect analysis. The results revealed that most of the test antibiotics did not show obvious synergy effect with the extracts against the test strains (data not shown).

### 2.6. Scanning Electron Microscope, SEM

**Figure 3:**
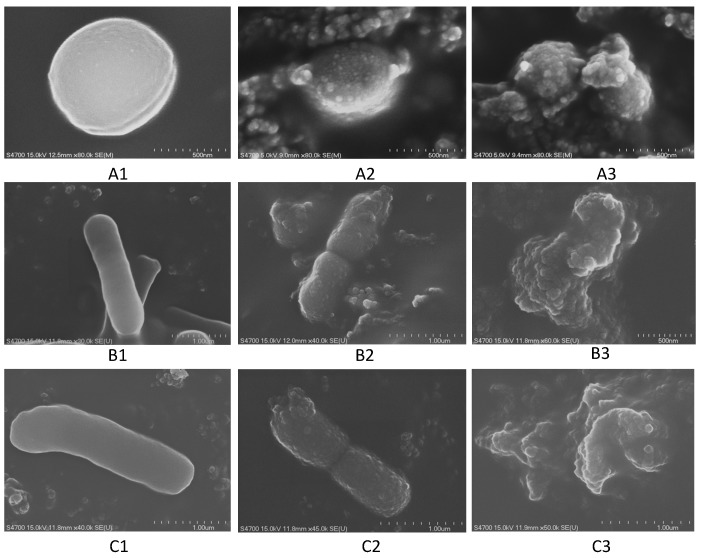
SEM observation of the cell morphology of the test microorganisms after treatment with the ethyl ether (EE) extracts from *Polygonum cuspidatum*. (**A**) strain *S. aureus* 335; (**B**) strain *A. baumannii* 3394; (**C**) strain *P. aeruginosa* 4016; 1 without adding the herbal extract; 2 adding half MIC dose of the EE extract; 3 adding two times MIC dose of the EE extract.

The cell morphology change of pathogens incubated with the EE extracts of *Polygonum cuspidatum* were observed by scanning electron microscopy (SEM). As shown in Figure 3, the results indicated that the cell morphology of test strains (*P. aeruginosa* 4016, *A. baumannii* 2260 and *S. aureus* 335 SA335) without being incubated with the EE extract displayed a complete and smooth surface (Figure 3A1–C1); while after incubating with a half MIC dose of the EE extract, the cell morphology of the strains appeared granular with the appearance of blisters (Figure 3A2–C2); if the strains were incubated with a 2-fold MIC dose of the EE extract, the cell-shapes of the bacteria were completely destroyed and the cells became atrophied and agglutinated (Figure 3A3–C3). This phenomenon suggests that the active substances from *Polygonum cuspidatum* may act on the cell membrane or extracellular proteins, resulting in the destruction of the bacterial cell growth.

## 3. Discussion

In this study, our results showed that both EE and EA extracts had greater antibacterial activities compared with hexane, chloroform and aqueous extracts of *P. cuspidatum*. This implies that the relatively medium polarity constituents have higher antimicrobial potential than the higher polarity or nonpolar constituents of *P. cuspidatum*. The antibacterial activities against all the clinical drug resistant pathogens suggest that the EE extracts from *P. cuspidatum* have a broad antimicrobial spectrum. *P. cuspidatum* is one of the important natural sources of resveratrol, which has been reported to possess numerous pharmacological properties and is responsible for many of the activities of *P. cuspidatum*, especially its anti-inflammatory activity [11], hepatoprotection [12], antibacterial activity [9,10], *etc*. Another phenolic substance, piceid, has also been found to be present at greater levels than its aglycone, resveratrol, in *P. cuspidatum* [9]. Previous literature has indicated that hydrolysis of this glycosylated derivative can occur in small intestine and liver, which would enhance the amount of available biologically active resveratrol [13]. According to the literature reports, we presume the active substances contributed to the antimicrobial activities could be resveratrol and piceid as well as other polyphenolic compounds. These phenolic substances all belong to the medium polarity substance class.

Based on the scanning electron microscopy observations, we found that the active substances of *P. cuspidatum* act on the bacterial cell membrane or outer membrane proteins, leading to the destruction of the bacterial cell growth. Similarly, other authors have reported that some plant products containing higher levels of polyphenol revealed significant antimicrobial activities [14,15,16]. The antimicrobial mechanism of action of polyphenols may be related to the inhibition of hydrolytic enzymes (proteases) or other interactions that inactivate microbial adhesions, cell envelope transport proteins and non-specific interactions with carbohydrates [17].

Plant and plant-based products have become crucial against various diseases or pathogen infections due to their lesser toxic and side effects. We have continuously for some time on the identification and characterization of antibacterial and antioxidant Chinese herbal medicines [18,19,20]. In the current study, we evaluated the antibacterial activities against clinical drug-resistant pathogens and studied the antibacterial mechanism to determine how the active ingredients of *Polygonum cuspidatum* can cause bacterial cell death. Although many researchers have focused on the investigation of the bioactivities of *Polygonum cuspidatum* [9,10,11,12,13,14,21,22], as far as we know, this study is the first demonstration of the remarkable antibacterial abilities of the ethyl ether fraction derived from *Polygonum cuspidatum* against nosocomial antibiotic resistant pathogens. Most importantly, the antibacterial mechanism of the active components from *Polygonum cuspidatum* was verified as due to action on the cell wall and cytoplasmic membrane. These results are in agreement with the findings of the authors of other plant studies [17].

## 4. Experimental Section

### 4.1. Plant Materials

Herbs used in this study were selected based on their usage as folk medicine and indications of the presence of compounds with antimicrobial properties. In this study, the root of *Polygonum cuspidatum* was purchased from local folk medicinal dealers and authorized by Kaohsiung Medicine University.

### 4.2. Test Bacterial Strains

A total of six clinical drug resistant strains, including *Staphylococcus aureus*, *Acinetobacter baumannii* and *P. aeruginosa*, were used in this study. The strains were isolated from patients’ blood or sputum during 2006–2008 and provided by the Chia-Yi Christian Hospital in Taiwan. Three reference strains, *Staphylococcus aureus* ATCC 6538P, *Acinetobacter baumannii* 19606 and *P. aeruginosa* ATCC 29260, were purchased from the Food Industry Research and Development Institute (Hsinchu, Taiwan).

### 4.3. Preparation of Crude Extracts

**Figure 4:**
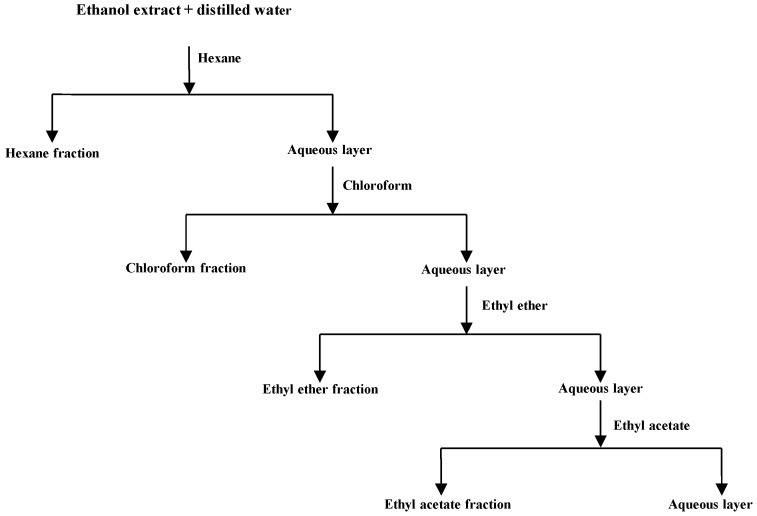
Fractionation of the alcoholic extracts from *Polygonum cuspidatum* by partition between immiscible solvents.

The dried herb (1.5 kg) was extracted with 4.5 L of 95% ethanol overnight by shaking in an incubator at 200 rpm and 37 °C. The ethanol extraction was repeated three times and the combined extracts were then filtered using Whatman filter paper No. 1 to remove insoluble debris. After filtration, the ethanol extract was dried by evaporation at a temperature of 40 °C. The crude extract were then successively partitioned with *n*-hexane, chloroform, ethyl ether and ethyl acetate based on solvent polarity, using the sequence of partition extraction shown in Figure 4. Each fraction was dried by evaporation, and the dried extracts were stored at 4 °C until the antimicrobial assay.

### 4.4. Antibacterial Activity Assay

The initial screening of the extracts for antibacterial activities was conducted by the disc diffusion method. The dried extracts were dissolved in dimethyl sulfoxide (DMSO) with a concentration of 0.1 g/mL. Paper discs (6 mm in diameter) were impregnated with 30 μL of herbal extracts and placed on cation-adjusted Mueller Hinton agar plates, which were inoculated with test organisms according to the standard protocol described by the National Committee of Clinical Laboratory Standards [23]. The plates were incubated at 37 °C and the diameters of the inhibition zones were measured after 18 h. Filter paper discs containing DMSO without any test compounds served as a control and no inhibition was observed. Additionally, for comparative purposes, tetracycline (15 mg/L, 30 μL) was used as a reference standard. Each assay was performed in triplicate and repeated three times.

### 4.5. Determination of Minimum Inhibitory Concentration (MIC)

The MIC of the crude extract and various fractions was determined by the agar dilution method, according to the NCCLS protocol [23] with some modification. The growth media, cation-adjusted Mueller-Hinton agar, was first prepared in the usual fashion and sterilized by autoclaving. The sterilized media were allowed to cool to 50 °C, and 10 mL of the molten agar was added to the test tubes, which contained different concentrations of the herbal extracts or the control substance (DMSO). The media and the test drugs were thoroughly mixed and poured into pre-labeled sterile Petri dishes on a level surface. The concentrations of the extracts used in these experiments ranged from 0.5 to 30 mg/mL. The densities of the cell suspensions of the respective microorganisms were adjusted to 5 × 10^6^ CFU/mL. The suspensions were transferred onto each plate and then incubated at 37 °C for 18 h. The lowest concentration which inhibited the growth of the respective microorganisms was taken as the MIC. All tests were carried out in triplicate.

### 4.6. Time-Killing Curve of the Ethyl Ether Extract

Three clinical drug resistant strains, Sa335, Ab2260 and Pa4016, were selected for time-killing curve analysis. The time-killing curve was determined by our previous study [19,20] with some modifications. The concentration of each antimicrobial agent in the cation-adjusted Mueller-Hinton broth was set at a concentration equal to double of the MIC level for the tested strain. A inoculate of *ca*. 5 × 10^5^ CFU/mL of bacteria harvested from the colonies grown overnight was used in these experiments. Aliquots (200 μL) of the cultures were taken at 0, 4, 8, 12, and 24 h and serially diluted in the Mueller-Hinton broth, and then plated on agar plates. Following 18 h of incubation, the number of colonies was counted to determine the total viable bacteria number. A cell culture without antimicrobial agent was assayed as the control.

### 4.7. Resistance Analysis of the Ethyl Ether Extract

The tested microorganisms (Sa335, Ab2260 and Pa4016) were sub-cultured in a sub-MIC concentration of the ethyl ether fraction for 10 consecutive days to investigate their ability to develop drug resistance. During the 10 days, culture purity was assured by Pyo (oxidase) test (Bright Glory Technology Inc., Kaohsiung, Taiwan) and the MIC of the subculture on day 11 was determined.

### 4.8. Combination Effect of the Ethyl Ether Extract with Antibiotics

Three clinical drug resistant strains, Sa335, Ab2260 and Pa4016, were selected for agent combination analysis by the disc diffusion method as described previously. The disc impregnated with active herbal fractions (100 mg/mL) was placed on the agar plate at a standard distance within the antibiotic discs. The plate was incubated at 37 °C and the pattern of inhibition zones was determined after 18 h. According to Matsuo’s method [24], bridging or confluent zones of inhibition between the discs of the sample and the antibiotics were considered to indicate synergism. The antibiotics used in this assay were erytromycin (25 mg/mL), gentamicin (15 mg/mL), tetracycline (7.5 mg/mL), spectinomycin (15 mg/mL), piperacillin G (30 mg/mL), kanamycin (15 mg/mL), amikacin (30 mg/mL), clindamycin (25 mg/mL), ampicillin (30 mg/mL), cephalosporinin (30 mg/mL), trimethoprim/sulfamethoxazole (30 mg/mL), and amoxillin (50 mg/mL).

### 4.9. Scanning Electron Microscope Observation

A single colony of the test strains was inoculated in 5 mL cation-adjusted Mueller-Hinton broth (with 50 μg/mL ampicillin), at 37 °C for 18 h, and then 100 μL of the culture suspension was inoculated in 5 mL cation-adjusted Mueller-Hinton broth containing the EE extract with a concentration of 0.5, 1, and 2 times of the MIC. The culture was incubated at 37 °C for 12 h; the cells were then harvested by centrifugation at 8000 rpm and prefixed with 5% glutaraldehyde (Sigma, St. Louis, MO, USA) in 0.1 M cacodylate buffer (pH 7.2) at 4 °C for 1.5 h. After being washed with the buffer, specimens were post fixed for 1 h with 1% osmium tetroxide in 0.1 M cacodylate buffer (pH 7.4) at 4 °C. The samples were dehydrated through a 30%, 50%, 70%, 90%, 100% ethanol series and dried at room temperature. The dried samples were finally treated by gold covered with catholic spraying; samples were examined by a HITACHI S-2700 Scanning Electron Microscope (Hitachi High-Tech, Tokyo, Japan).

### 4.10. Statistical Analysis

The experimental results were expressed as mean ± standard deviation (SD) of three replicates. Statistical significance was determined by student’s *t*-test. *p*-value < 0.05 was considered as significant.

## 5. Conclusions

The finding of this study showed among all the fractions of the ethanol extract of *Polygonum cuspidatum* the EE-soluble fraction possessed the highest antibacterial activity and a broad antimicrobial spectrum against the test pathogens. The antibacterial mechanisms of the EE extract’s toxicity against microbes may be due to action against the cell membrane or extracellular proteins, resulting in the destruction of the bacterial cell by apoptosis. The EE extracts from *Polygonum cuspidatum* may provide a promising antimicrobial agent for therapeutic applications against nosocomial drug-resistant bacteria. Further studies are needed to identify the active components a of the EE extract of *Polygonum cuspidatum*. In addition, the *in vitro* studies for investigation of the effect of EE extract on normal human cell lines are suggested prior to verify the bioactivity in animal models.
